# Tri-(2-ethylhexyl) trimellitate – Determination of 1-MEHTM, 2-MEHTM, 5OH-1-MEHTM, 5OH-2-MEHTM, 5cx-1-MEPTM, and 5cx-2-MEPTM in urine by LC-MS/MS: Biomonitoring Method – Translation of the German version from 2025

**DOI:** 10.34865/bi331931e10_4or

**Published:** 2025-12-22

**Authors:** Laura Kuhlmann, Elisabeth Eckert, Christine Höllerer-Mittelmaier, Thomas Göen, Veronika Spindler, Gerhard Scherer, Craig Sams, Kate Jones, Andrea Hartwig, MAK Commission

**Affiliations:** 1 Friedrich-Alexander-Universität Erlangen-Nürnberg. Institute and Outpatient Clinic of Occupational, Social, and Environmental Medicine Henkestraße 9–11 91054 Erlangen Germany; 2 ABF Analytisch-biologisches Forschungslabor GmbH Semmelweisstraße 5 82152 Planegg Germany; 3 Health and Safety Executive (HSE) Science and Research Centre Harpur Hill SK17 9JN Buxton (Derbyshire) United Kingdom; 4 Institute of Applied Biosciences. Department of Food Chemistry and Toxicology. Karlsruhe Institute of Technology (KIT) Adenauerring 20a, Building 50.41 76131 Karlsruhe Germany; 5 Permanent Senate Commission for the Investigation of Health Hazards of Chemical Compounds in the Work Area. Deutsche Forschungsgemeinschaft, Kennedyallee 40, 53175 Bonn, Germany. Further information: Permanent Senate Commission for the Investigation of Health Hazards of Chemical Compounds in the Work Area | DFG

**Keywords:** TEHTM, biomonitoring, urine, LC-MS/MS

## Abstract

The working group “Analyses in Biological Materials” of the German Senate Commission for the Investigation of Health Hazards of Chemical Compounds in the Work Area (MAK Commission) developed and verified this biomonitoring method for the measurement of six specific metabolites of the plasticiser tri‑(2‑ethylhexyl) trimellitate (TEHTM) in urine. Specifically, this method determines two monoester isomers as primary hydrolysis products of TEHTM, 1‑mono-(2‑ethylhexyl) trimellitate (1‑MEHTM) and 2‑mono-(2‑ethylhexyl) trimellitate (2‑MEHTM), as well as the oxidatively formed secondary derivatives, namely 1‑mono-(2‑ethyl-5‑hydroxyhexyl) trimellitate (5OH‑1‑MEHTM), 2‑mono-(2‑ethyl-5‑hydroxyhexyl) trimellitate (5OH‑2‑MEHTM), 1‑mono-(2‑ethyl-5‑carboxypentyl) trimellitate (5cx‑1‑MEPTM), and 2‑mono-(2‑ethyl-5‑carboxypentyl) trimellitate (5cx‑2‑MEPTM). Determination is carried out after enzymatic hydrolysis of the urine sample as well as enrichment of the analytes by online SPE. Via integrated, automatic column-switching, the analytes are transferred onto the analytical column in backflush mode, separated by liquid chromatography, and quantified by tandem mass spectrometry. Calibration is performed using calibration standards prepared in pooled urine and processed analogously to the samples to be analysed. The following isotope-labelled substances are added to the urine samples as internal standards: D_5_‑1‑MEHTM, D_5_‑2‑MEHTM, D_5_‑5OH‑1‑MEHTM, D_5_‑5cx‑1‑MEPTM, and D_5_‑5cx‑2‑MEPTM. The method provides reliable and accurate analytical results, as shown by the good precision data with standard deviations no greater than 8%. Good accuracy data were obtained with mean relative recoveries in the range of 97–109%. The method is both selective and sensitive, and provides quantitation limits in the range of 0.04–0.12 μg/l.

## 1 Characteristics of the method

**Table:** 

**Matrix**	Urine		
**Analytical principle**	Liquid chromatography with tandem mass spectrometry (LC‑MS/MS)		
**Parameters and corresponding hazardous substance**			
**Hazardous substance**	**CAS No.**	**Parameter**	**CAS No.**
Tri-(2‑ethylhexyl) trimellitate (TEHTM)	3319-31-1	1‑Mono-(2‑ethylhexyl) trimellitate (1‑MEHTM)	61137-09-5
		2‑Mono-(2‑ethylhexyl) trimellitate (2‑MEHTM)	63468-08-6
		1‑Mono-(2‑ethyl-5‑hydroxyhexyl) trimellitate (5OH‑1‑MEHTM)	2306733-44-6
		2‑Mono-(2‑ethyl-5‑hydroxyhexyl) trimellitate (5OH‑2‑MEHTM)	2306733-45-7
		1‑Mono-(2‑ethyl-5‑carboxypentyl) trimellitate (5cx‑1‑MEPTM)	2306733-48-0
		2‑Mono-(2‑ethyl-5‑carboxypentyl) trimellitate (5cx‑2‑MEPTM)	2306733-49-1

### Reliability criteria

#### 1‑MEHTM

**Table:** 

Within-day precision:	Standard deviation (rel.)	*s*_w_ = 3.0% or 6.3%
	Prognostic range	*u* = 6.8% or 14.3%
	at a spiked concentration of 0.24 μg or 1.20 μg 1‑MEHTM per litre of urine and n = 10 determinations	
Day-to-day precision:	Standard deviation (rel.)	*s*_w_ = 5.2% or 6.6%
	Prognostic range	*u* = 12.3% or 15.9%
	at a spiked concentration of 0.24 μg or 1.20 μg 1‑MEHTM per litre of urine and n = 8 determinations	
Accuracy:	Recovery (rel.)	*r* = 100% or 99.0%
	at a spiked concentration of 0.24 μg or 1.20 μg 1‑MEHTM per litre of urine and n = 8 determinations	
Limit of detection:	0.01 μg 1‑MEHTM per litre of urine	
Limit of quantitation:	0.04 μg 1‑MEHTM per litre of urine	

#### 2‑MEHTM

**Table:** 

Within-day precision:	Standard deviation (rel.)	*s*_w_ = 4.3% or 6.3%
	Prognostic range	*u* = 9.7% or 14.3%
	at a spiked concentration of 0.25 μg or 1.27 μg 2‑MEHTM per litre of urine and n = 10 determinations	
Day-to-day precision:	Standard deviation (rel.)	*s*_w_ = 4.0% or 7.3%
	Prognostic range	*u* = 9.5% or 17.3%
	at a spiked concentration of 0.25 μg or 1.27 μg 2‑MEHTM per litre of urine and n = 8 determinations	
Accuracy:	Recovery (rel.)	*r* = 104% or 96.8%
	at a spiked concentration of 0.25 μg or 1.27 μg 2‑MEHTM per litre of urine and n = 8 determinations	
Limit of detection:	0.02 μg 2‑MEHTM per litre of urine	
Limit of quantitation:	0.07 μg 2‑MEHTM per litre of urine	

#### 5OH‑1‑MEHTM

**Table:** 

Within-day precision:	Standard deviation (rel.)	*s*_w_ = 5.0% or 4.0%
	Prognostic range	*u* = 11.3% or 9.0%
	at a spiked concentration of 0.26 μg or 1.28 μg 5OH‑1‑MEHTM per litre of urine and n = 10 determinations	
Day-to-day precision:	Standard deviation (rel.)	*s*_w_ = 5.0% or 3.7%
	Prognostic range	*u* = 11.8% or 8.8%
	at a spiked concentration of 0.26 μg or 1.28 μg 5OH‑1‑MEHTM per litre of urine and n = 8 determinations	
Accuracy:	Recovery (rel.)	*r* = 98.9% or 99.0%
	at a spiked concentration of 0.26 μg or 1.28 μg 5OH‑1‑MEHTM per litre of urine and n = 8 determinations	
Limit of detection:	0.02 μg 5OH‑1‑MEHTM per litre of urine	
Limit of quantitation:	0.07 μg 5OH‑1‑MEHTM per litre of urine	

#### 5OH‑2‑MEHTM

**Table:** 

Within-day precision:	Standard deviation (rel.)	*s*_w_ = 8.1% or 5.5%
	Prognostic range	*u* = 18.3% or 12.4%
	at a spiked concentration of 0.25 μg or 1.24 μg 5OH‑2‑MEHTM per litre of urine and n = 10 determinations	
Day-to-day precision:	Standard deviation (rel.)	*s*_w_ = 5.9% or 2.7%
	Prognostic range	*u* = 14.0% or 6.3%
	at a spiked concentration of 0.25 μg or 1.24 μg 5OH‑2‑MEHTM per litre of urine and n = 8 determinations	
Accuracy:	Recovery (rel.)	*r* = 104% or 109%
	at a spiked concentration of 0.25 μg or 1.24 μg 5OH‑2‑MEHTM per litre of urine and n = 8 determinations	
Limit of detection:	0.04 μg 5OH‑2‑MEHTM per litre of urine	
Limit of quantitation:	0.12 μg 5OH‑2‑MEHTM per litre of urine	

#### 5cx‑1‑MEPTM

**Table:** 

Within-day precision:	Standard deviation (rel.)	*s*_w_ = 2.4% or 6.0%
	Prognostic range	*u* = 5.4% or 13.6%
	at a spiked concentration of 0.24 μg or 1.20 μg 5cx‑1‑MEPTM per litre of urine and n = 10 determinations	
Day-to-day precision:	Standard deviation (rel.)	*s*_w_ = 6.0% or 4.8%
	Prognostic range	*u* = 14.2% or 11.4%
	at a spiked concentration of 0.24 μg or 1.20 μg 5cx‑1‑MEPTM per litre of urine and n = 8 determinations	
Accuracy:	Recovery (rel.)	*r* = 105% or 109%
	at a spiked concentration of 0.24 μg or 1.20 μg 5cx‑1‑MEPTM per litre of urine and n = 8 determinations	
Limit of detection:	0.01 μg 5cx‑1‑MEPTM per litre of urine	
Limit of quantitation:	0.05 μg 5cx‑1‑MEPTM per litre of urine	

#### 5cx‑2‑MEPTM

**Table:** 

Within-day precision:	Standard deviation (rel.)	*s*_w_ = 4.8% or 6.0%
	Prognostic range	*u* = 10.9% or 13.6%
	at a spiked concentration of 0.24 μg or 1.21 μg 5cx‑2‑MEPTM per litre of urine and n = 10 determinations	
Day-to-day precision:	Standard deviation (rel.)	*s*_w_ = 4.8% or 5.6%
	Prognostic range	*u* = 11.4% or 13.2%
	at a spiked concentration of 0.24 μg or 1.21 μg 5cx‑2‑MEPTM per litre of urine and n = 8 determinations	
Accuracy:	Recovery (rel.)	*r* = 99.4% or 100%
	at a spiked concentration of 0.24 μg or 1.21 μg 5cx‑2‑MEPTM per litre of urine and n = 8 determinations	
Limit of detection:	0.01 μg 5cx‑2‑MEPTM per litre of urine	
Limit of quantitation:	0.04 μg 5cx‑2‑MEPTM per litre of urine	

## 2 General information on TEHTM

Tri‑(2‑ethylhexyl) trimellitate (TEHTM) is a plasticiser which is used in particular as a substitute for di‑(2‑ethylhexyl) phthalate (DEHP) (Bourdeaux et al. 2016; SCENIHR 2015; Van Vliet et al. 2011). Compared to DEHP, TEHTM is characterised by lower toxicity as well as a significantly lower migration rate in contact media (Eckert et al. 2016). To date, TEHTM has been primarily used in medical products made of soft PVC, particularly for bags and tubing intended for use with blood, plasma, infusions, or artificial nutrition. The proportion of the plasticisers in PVC material is usually 20–40% of the product’s total weight (Bernard et al. 2014; Green et al. 2005). Since these plasticisers are not chemically bound to PVC, they might migrate into the contact medium, e.g. blood. As a result, patients may be directly exposed to TEHTM, representing the most important route of exposure to this substance. The available cellular and animal studies on TEHTM were mostly performed in direct comparison to DEHP. In these studies, a considerably lower ­toxicity could be determined for TEHTM when compared with DEHP (Eljezi et al. 2017; Hodgson 1987; Kambia et al. 2004; Ohashi et al. 2005). As no human data were available, the human metabolism of TEHTM was investigated for the first time in an *in vivo* study as part of the BMU‑VCI project (Höllerer et al. 2018 a). The method hereby applied has been published internationally (Höllerer et al. 2018 b).

**Fig.1:**
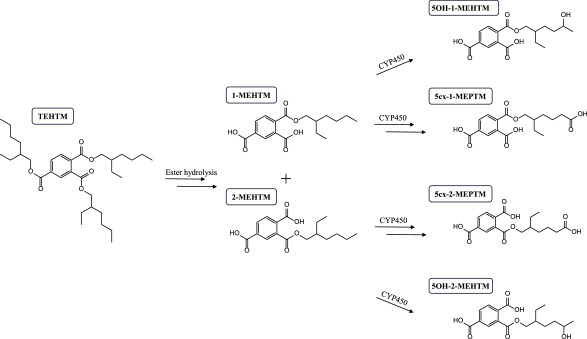
Metabolism scheme for TEHTM, highly simplified, according to Höllerer et al. (2018 a) and Kuhlmann et al. (2021)

The results of the *invivo* study show that TEHTM is absorbed after oral ingestion and is first selectively cleaved to the diester isomers 1,2‑di‑(2‑ethylhexyl) trimellitate (1,2‑DEHTM) and 2,4‑di‑(2‑ethylhexyl) trimellitate (2,4‑DEHTM). These isomers are then further broken down into the monoester isomers 1‑MEHTM and 2‑MEHTM. In contrast, the diester isomer 1,4‑di‑(2‑ethylhexyl) trimellitate (1,4‑DEHTM) and the monoester 4‑MEHTM could not be detected in the blood. All three monoester isomers – 1‑MEHTM, 2‑MEHTM, and 4‑MEHTM – could be detected in the urine, whereby 4‑MEHTM was only found in very small amounts. With respect to the relevant monoesters 1‑MEHTM and 2‑MEHTM, secondary metabolites (oxidation at the side chain) were investigated which could also be detected in the urine of the exposed persons. A metabolism scheme for TEHTM could be created based on these results and is depicted in Figure 1. In general, it could be shown that the metabolism of TEHTM and the excretion of its metabolites in urine is a relatively slow process in humans, as some metabolites were still detectable in the urine 72 h after exposure (Höllerer et al. 2018 a). For five of the urinary metabolites that can be determined with this method, biphasic elimination kinetics were observed. Half-lives of between 4 and 6 hours and between 10 and 33 hours, respectively, were calculated for excretion with the urine (Höllerer et al. 2018 a). For 5cx‑2‑MEPTM, monophasic elimination kinetics with a half-life of 17 h have been reported. In total, about 6% of the orally administered TEHTM dose was found in the urine as metabolites within 72 h, whereby 2‑MEHTM was the main metabolite, followed by 5cx‑1‑MEPTM, 5OH‑1‑MEHTM, 5OH‑2‑MEHTM, and 1‑MEHTM; as a result, these metabolites can likewise be recommended as biomarkers for the human biomonitoring of TEHTM. Accordingly, in the method described herein, the metabolites of the original method from Höllerer et al. (2018 a) were limited to these six metabolites (Kuhlmann et al. 2021).

## 3 General principles

The method described herein enables the measurement of six specific metabolites of the plasticiser TEHTM in urine. The analytes consist of two monoester isomers as primary hydrolysis products of TEHTM (1‑MEHTM and 2‑MEHTM) as well as the oxidatively formed secondary derivatives (5OH‑1‑MEHTM, 5OH‑2‑MEHTM, 5cx‑1‑MEPTM, and 5cx‑2‑MEPTM). Determination is carried out after enzymatic hydrolysis of the urine sample as well as enrichment of the analytes by online SPE. Via integrated, automatic column-switching, the analytes are transferred onto the analytical column in backflush mode, separated by liquid chromatography, and quantified by tandem mass spectrometry. Calibration is performed using calibration standards prepared in pooled urine and processed analogously to the ­samples to be analysed. The following isotope-labelled substances are added to the urine samples as internal standards (ISTDs): D_5_‑1‑MEHTM, D_5_‑2‑MEHTM, D_5_‑5OH‑1‑MEHTM, D_5_‑5cx‑1‑MEPTM, and D_5_‑5cx‑2‑MEPTM.

## 4 Equipment, chemicals, and solutions

### 4.1 Equipment

- UPLC system (e.g. ACQUITY UPLC H‑Class System, Waters GmbH, Eschborn, Germany) with a quaternary pump (e.g. ACQ H‑Class QSM Plus, Waters GmbH, Eschborn, Germany), a binary pump (e.g. UPLC Binary SOL MGR, Waters GmbH, Eschborn, Germany), an autosampler (e.g. ACQ H‑Class FTN‑H Plus, Waters GmbH, Eschborn, Germany), and a column manager (e.g. ACQUITY UPLC CM‑A, Waters GmbH, Eschborn, Germany)
- Triple-quadrupole mass spectrometer (e.g. Xevo TQ‑XS, Waters GmbH, Eschborn, Germany)
- Data-evaluation software (e.g. MassLynx 4.2, TargetLynx XS, Waters GmbH, Eschborn, Germany)
- Enrichment column (e.g. No. 186005233, XBridge^®^ BEH C8 Direct Connect HP (30 mm × 2.1 mm, 10 μm), restricted access material (RAM) phase, Waters GmbH, Eschborn, Germany)
- Precolumn for the LC column (e.g. No. 9309A0252, Raptor^TM^ biphenyl with EXP^®^ Direct Connect precolumn cartridge holder (5 mm × 2.1 mm, 2.7 μm), Restek Corporation, Bellefonte, PA, USA)
- Analytical UPLC column (e.g. No. 9309212, core-shell Raptor^TM^ biphenyl (100 mm × 2.1 mm, 1.8 μm), Restek ­Cor­poration, Bellefonte, PA, USA)
- In-line filter (e.g. No. 205000343, ACQUITY Column In-Line Filter Waters Critical Clean^TM^, Waters GmbH, Esch­born, Germany)
- Laboratory centrifuge (e.g. Megafuge^TM^, Heraeus Holding GmbH, Hanau, Germany)
- Vortex shaker (e.g. Vortex 2, IKA‑Werke GmbH & Co. KG, Staufen, Germany)
- Thermostatted drying oven (e.g. Memmert GmbH + Co. KG, Schwabach, Germany)
- 1.8‑ml threaded vials, clear, with 8‑mm screw caps with PTFE‑lined septa (e.g. No. 548‑0018 and No. 548‑0024, VWR International GmbH, Darmstadt, Germany)
- 2‑ml threaded vials, certified for LC‑MS, clear, with 12‑mm screw caps with pre-perforated PTFE-lined septa (e.g. No. 600000668CV, Waters GmbH, Eschborn, Germany)
- Microlitre pipettes, variably adjustable between volumes of 10 μl–100 μl as well as 100 μl–1000 μl (e.g. Eppen­dorf AG, Hamburg, Germany) and matching pipette tips (e.g. FinnTip^TM^, Thermo Fisher Scientific^TM^, Life Techno­logies GmbH, Darmstadt, Germany)
- Multipettes^TM^ (e.g. M4, Eppendorf AG, Hamburg, Germany)
- Various volumetric flasks, measurement cylinders, and glass beakers (e.g. DURAN^®^, Schott AG, Mainz, Germany)
- Bottles for HPLC mobile phases, 1 l (e.g. Waters GmbH, Eschborn, Germany or DURAN^®^, Schott AG, Mainz, Germany)
- Magnetic stirrer (e.g. RSM‑10HS, Phoenix Instrument GmbH, Garbsen, Germany)
- pH meter (e.g. Mettler-Toledo GmbH, Gießen, Germany)
- Urine cups (plasticiser-free, e.g. made of polypropylene, Sarstedt AG & Co. KG, Nümbrecht, Germany)

### 4.2 Chemicals

Unless otherwise specified, all chemicals must be a minimum of *pro analysi* grade.

- 1‑MEHTM (e.g. No. B190025, Toronto Research Chemicals Inc., Toronto, Canada)
- 2‑MEHTM (e.g. No. B190020, Toronto Research Chemicals Inc., Toronto, Canada)
- 5OH‑1‑MEHTM, 99% (e.g. custom synthesis, Max Planck Institute for Biophysical Chemistry, Faculty of Synthetic Organic Chemistry, Göttingen, Germany)
- 5OH‑2‑MEHTM, 95% (e.g. custom synthesis, Max Planck Institute for Biophysical Chemistry, Faculty of Synthetic Organic Chemistry, Göttingen, Germany)
- 5cx‑1‑MEPTM, 95% (e.g. custom synthesis, Max Planck Institute for Biophysical Chemistry, Faculty of Synthetic Organic Chemistry, Göttingen, Germany)
- 5cx‑2‑MEPTM, 95% (e.g. custom synthesis, Max Planck Institute for Biophysical Chemistry, Faculty of Synthetic Organic Chemistry, Göttingen, Germany)
- Mixture of D_5_‑1‑MEHTM and D_5_‑2‑MEHTM (D_5_‑1‑MEHTM ∶ D_5_‑2‑MEHTM, 40 ∶ 60, w/w), 95% (e.g. custom ­synthesis, Max Planck Institute for Biophysical Chemistry, Faculty of Synthetic Organic Chemistry, Göttingen, Germany)
- D_5_‑5OH‑1‑MEHTM, 95% (e.g. custom synthesis, Max Planck Institute for Biophysical Chemistry, Faculty of Synthetic Organic Chemistry, Göttingen, Germany)
- D_5_‑5cx‑1‑MEPTM, 95% (e.g. custom synthesis, Max Planck Institute for Biophysical Chemistry, Faculty of Synthetic Organic Chemistry, Göttingen, Germany)
- D_5_‑5cx‑2‑MEPTM, 97% (e.g. custom synthesis, Max Planck Institute for Biophysical Chemistry, Faculty of Synthetic Organic Chemistry, Göttingen, Germany)
- Acetic acid (100%, glacial acetic acid) (e.g. No. 1.00063, Merck KGaA, Darmstadt, Germany)
- Acetonitrile (e.g. No. 83640, HiPerSolv CHROMANORM^®^ for LC‑MS analysis, VWR International GmbH, Darmstadt, Germany)
- Ammonium acetate (e.g. No. 1.01116, Merck KGaA, Darmstadt, Germany)
- Formic acid (e.g. No. 84865, HiPerSolv CHROMANORM^®^ for LC‑MS analysis, VWR International GmbH, Darmstadt, Germany)
- Methanol (e.g. No. 83638, HiPerSolv CHROMANORM^®^ for LC‑MS analysis, VWR International GmbH, Darmstadt, Germany)
- 2‑Propanol (e.g. No. 84881, HiPerSolv CHROMANORM^®^ for LC‑MS analysis, VWR International GmbH, Darmstadt, Germany)
- Ultra-pure water (e.g. No. 83645, HiPerSolv CHROMANORM^®^ for LC‑MS analysis, VWR International GmbH, Darmstadt, Germany)
- *β*‑Glucuronidase from* Escherichia coli* K12 (e.g. No. 03707598001, Roche Diagnostics Deutschland GmbH, Mannheim, Germany)
- Argon 5.0 (e.g. Linde GmbH, Pullach, Germany)
- Nitrogen 5.0 (e.g. Linde GmbH, Pullach, Germany)
- Native urine from volunteers with the lowest possible background levels of TEHTM metabolites

### 4.3 Solutions

- Eluent A1 (methanol)
  Methanol is used as Eluent A1.
- Eluent B1 (water ∶ methanol ∶ formic acid (80 ∶ 20 ∶ 0.1; v/v/v))
  In a measurement cylinder, 800 ml of ultra-pure water and 200 ml of methanol are separately measured and transferred into an empty mobile-phase bottle; 1 ml of formic acid is then added. The mobile-phase bottle is sealed and thoroughly shaken.
- Eluent A2 (water ∶ formic acid (100 ∶ 0.1; v/v))
  In a measurement cylinder, 1000 ml of ultra-pure water are measured and transferred into an empty mobile-phase bottle; 1 ml of formic acid is then added. The mobile-phase bottle is sealed and thoroughly shaken.
- Eluent B2 (acetonitrile ∶ formic acid (100 ∶ 0.1; v/v))
  In a measurement cylinder, 1000 ml of acetonitrile are measured and transferred into an empty mobile-phase bottle; 1 ml of formic acid is then added. The mobile-phase bottle is sealed and thoroughly shaken.

When stored at room temperature, the eluents are stable for at least three months.

- Ammonium acetate buffer (1 mol/l, pH 6.5)
  Exactly 19.27 g of ammonium acetate are weighed into a 250‑ml glass beaker and dissolved in about 200 ml of ultra-pure water while stirring. Subsequently, the pH value is adjusted to pH 6.5 by adding glacial acetic acid. The solution is transferred to a 250‑ml volumetric flask and made up to the mark with ultra-pure water.

The solution is stable for at least three months when refrigerated at 4 °C.

### 4.4 Internal standards (ISTDs)

- ISTD stock solutions (1000 mg/l)
  10 mg of the mixture of D_5_‑1‑MEHTM and D_5_‑2‑MEHTM as well as 10 mg each of D_5_‑5OH‑1‑MEHTM, D_5_‑5cx‑1‑MEPTM, and D_5_‑5cx‑2‑MEPTM are weighed exactly into separate 10‑ml volumetric flasks and dissolved in methanol. The flasks are then made up to the mark with methanol.
- ISTD working solution
  Approximately 5 ml of methanol are placed in a 20‑ml volumetric flask. Subsequently, 50 μl of the stock solution of D_5_‑1‑MEHTM and D_5_‑2‑MEHTM are added as well as 20 μl of each of the stock solutions of D_5_‑5OH‑1‑MEHTM, D_5_‑5cx‑1‑MEPTM, and D_5_‑5cx‑2‑MEPTM. The flask is then made up to the mark with methanol.
- ISTD spiking solution
  In a 50‑ml volumetric flask, 5 ml of the ISTD working solution are diluted with methanol. This ISTD ­spiking solution contains concentrations of 100 μg of D_5_‑1‑MEHTM/l, 150 μg of D_5_‑2‑MEHTM/l, and 100 μg each of D_5_‑5OH‑1‑MEHTM, D_5_‑5cx‑1‑MEPTM, and D_5_‑5cx‑2‑MEPTM/l.

The stock solutions as well as the working solution and the spiking solution are stored at −20 °C and are stable under these conditions for at least six months.

### 4.5 Calibration standards

- Stock solutions (1000 mg/l)
  Methanolic stock solutions are used, in which the respective analyte concentration is 1000 mg/l.
- Spiking solution I (100 μg/l)
  About 50 ml of ultra-pure water are placed in a 200‑ml volumetric flask. Subsequently, 20 μl of each stock solution are added. The flask is made up to the mark with ultra-pure water.
- Spiking solution II (10 μg/l)
  About 5 ml of ultra-pure water are placed in a 20‑ml volumetric flask and 2 ml of Spiking solution I are added. The flask is made up to the mark with ultra-pure water.

The stock and spiking solutions are stored at −20 °C and are stable under these conditions for at least six months.

For calibration, pooled urine from persons with no occupational exposure to plasticisers is used. Any urinary background levels of the analytes are subtracted. For the preparation of the calibration standards, urine is mixed with the spiking solutions according to the pipetting scheme presented in Table 1. The pooled urine is included as a blank. Fresh calibration standards are prepared for each analytical run.

**Tab.1:** Pipetting scheme for the preparation of calibration standards for the determination of TEHTM metabolites in urine

Calibration standard	Spiking solution I [μl]	Spiking solution II [μl]	Pooled urine [μl]	Analyte concentration [μg/l]
0	–	–	1000	0.0
1	–	10	990	0.1
2	–	20	980	0.2
3	–	50	950	0.5
4	10	–	990	1.0
5	20	–	980	2.0
6	50	–	950	5.0

## 5 Specimen collection and sample preparation

### 5.1 Specimen collection

The urine samples are collected in sealable plastic containers (free of plasticisers, e.g. made of polypropylene) and frozen at −20 °C. When stored in this manner, the urine is stable for at least one year.

### 5.2 Sample preparation

Prior to analysis, the samples are thawed at room temperature and thoroughly mixed. An aliquot of 1 ml is taken from each urine sample and transferred into a 1.8‑ml threaded vial; 200 μl of ammonium acetate buffer, 50 μl of the ISTD spiking solution, and 10 μl of the *β*‑glucuronidase solution are then added. The samples are thoroughly mixed using a vortex mixer and are incubated in a drying oven at 37 °C for 2 h. The samples are again thoroughly mixed and finally stored at −20 °C until frozen (at least 1 h, ideally overnight) to interrupt hydrolysis.

The samples are then thawed and centrifuged at 3000 × *g* for 10 min. Using narrow pipette tips (e.g. FinnTip^TM^, Thermo Fisher Scientific^TM^, Life Technologies GmbH, Darmstadt, Germany), the supernatants are transferred into new ­threaded vials suitable for instrumental analysis (2‑ml threaded vials from Waters GmbH, Eschborn, Germany), which are then sealed. For analysis, 30 μl of each sample are injected into the LC‑MS/MS system.

## 6 Operational parameters

**Fig.2:**
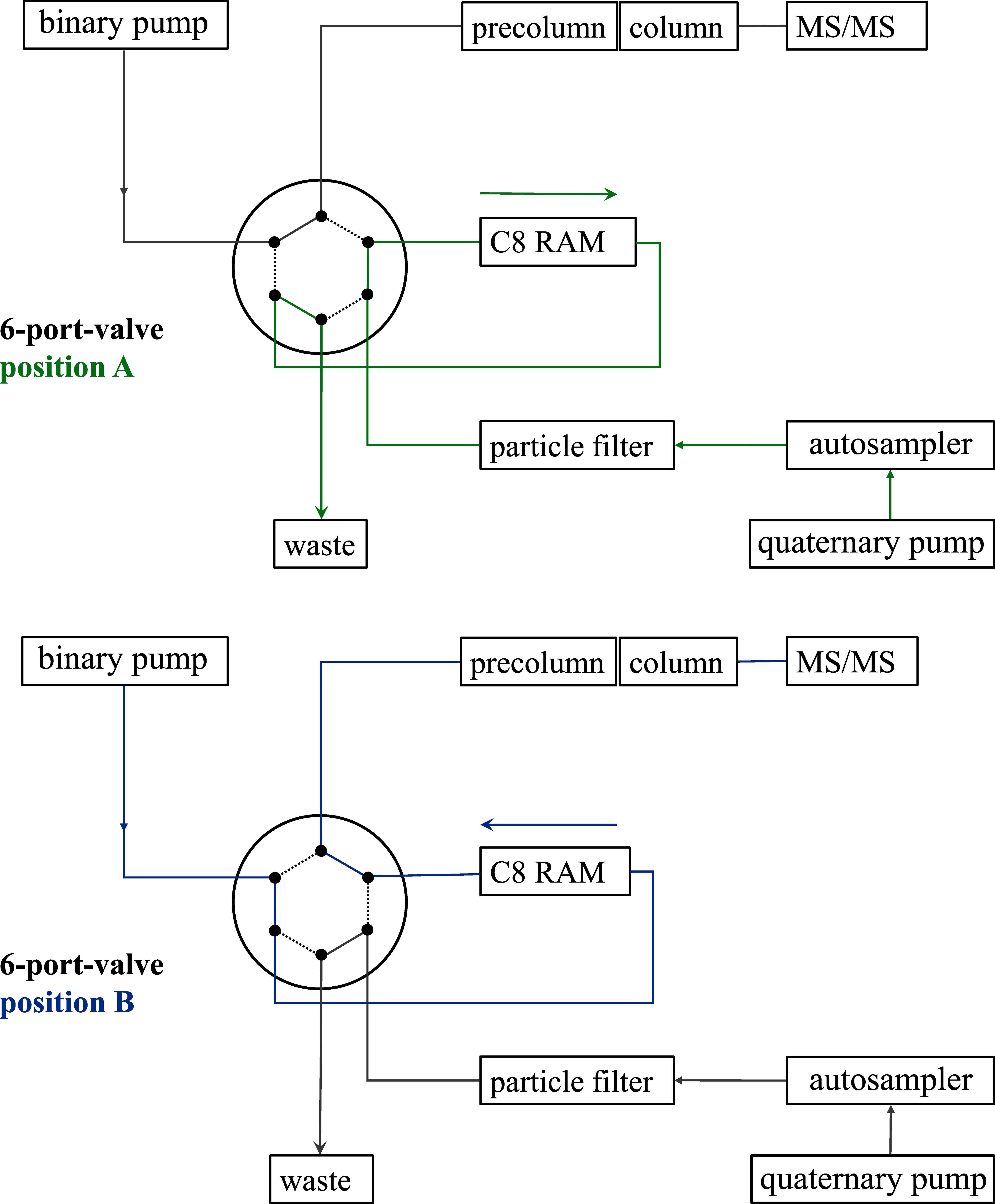
Schematic depiction of the LC‑MS/MS and illustration of switch positions A (green) and B (blue) of the six-port valve

Analytical determination is carried out using a device combination comprised of a UPLC system, a ten-port switching valve, and a tandem mass spectrometer. Two HPLC pumps are required for sample preparation and separation (quaternary pump P1 and binary pump P2). The two chromatographic columns (RAM phase and analytical column) are coupled via a six-port switching valve (six ports of the ten-port switching valve are used, see Figure 2).

### 6.1 Liquid chromatography

**Table:** 

Enrichment column:	XBridge^®^ BEH C8 Direct Connect HP (30 mm × 2.1 mm, 10 μm)	
Precolumn:	Raptor^TM^ biphenyl (5 mm × 2.1 mm, 2.7 μm)	
Analytical column:	Core-shell Raptor^TM^ biphenyl (100 mm × 2.1 mm, 1.8 μm)	
Separation principle:	Reversed phase	
Mobile phase of quaternary pump P1:	Eluent A1:	Methanol
	Eluent B1:	Water ∶ methanol ∶ formic acid (80 ∶ 20 ∶ 0.1; v/v/v)
Mobile phase of binary pump P2:	Eluent A2:	Water ∶ formic acid (100 ∶ 0.1; v/v)
	Eluent B2:	Acetonitrile ∶ formic acid (100 ∶ 0.1; v/v)
Flow rate of quaternary pump P1:	0.5 ml/min; see Table 2	
Flow rate of binary pump P2:	0.3 ml/min; see Table 3	
Programme of switching valve:	see Table 4	

**Tab.2:** Gradient programme of quaternary pump P1 for the online enrichment of the analytes on the RAM phase

Time [min]	Flow rate [ml/min]	Eluent A1 [%]	Eluent B1 [%]
0	0.5	0	100
6.0	0.5	0	100
6.1	0.5	100	0
14.0	0.5	100	0
14.5	0.5	50	50
15.0	0.5	0	100
18.0	0.5	0	100

**Tab.3:** Gradient programme of binary pump P2 for the chromatographic separation of the analytes

Time [min]	Flow rate [ml/min]	Eluent A2 [%]	Eluent B2 [%]
0	0.3	75	25
2.0	0.3	75	25
3.0	0.3	70	30
8.0	0.3	70	30
9.0	0.3	45	55
11.0	0.3	45	55
12.0	0.3	10	90
15.0	0.3	10	90
16.0	0.3	75	25
18.0	0.3	75	25

**Tab.4:** Switching programme of the six-port valve (see also Figure 2)

Time [min]	Switch position	Description
0–4.0	A	Enrichment of analytes on RAM phase via pump P1, equilibration of analytical column
4.0–6.0	B	Transfer of analytes onto analytical column via pump P2, chromatographic separation of analytes
6.0–11.0	A	Reconditioning of RAM phase via pump P1, chromatographic separation of analytes via pump P2
11.0–14.0	B	Flushing of RAM phase on analytical column via pump P2, chromatographic separation of analytes
14.0–18.0	A	Reconditioning of RAM phase via pump P1 and of analytical column via pump P2

It is recommended to add a particle filter before the RAM phase in order to avoid blockages. All other parameters must be optimised according to manufacturer specifications.

### 6.2 Tandem mass spectrometry

**Table:** 

Ionisation:	Electrospray, negative (ESI−)
Ion-spray voltage:	−4500 V
Capillary voltage:	1 kV
Source temperature:	150 °C
Desolvation temperature:	500 °C
Dwell time:	25 ms
Nebuliser gas:	Nitrogen, 700 kPa
Collision gas:	Argon, 0.15 ml/min
Detection mode:	Multiple Reaction Monitoring (MRM)

The instrument-specific parameters must be ascertained and adjusted by the user for the MS/MS system used. The device-specific parameters given in this section have been determined and optimised for the system used during method development.

## 7 Analytical determination

For the analytical determination of the urine samples processed according to Section 5.2, an aliquot of 30 μl is injected into the LC‑MS/MS system by the autosampler. After online enrichment and chromatographic separation, the analytes are identified by their retention times and characteristic mass transitions (see Table 5). Ultra-pure water, serving as a blank, as well as at least two quality-control samples are included as part of each analytical run. Isomers which exhibit identical mass-spectrometric fragmentation are distinguished and identified by their retention times. Analytes with similar retention times are distinguished by their characteristic mass transitions.

**Tab.5:** Mass transitions of the analytes and ISTDs, retention times, and further parameter-specific settings

Analyte or ISTD	Precursor ion (*m/z*)	Product ion (*m/z*)	Cone voltage [V]	Collision energy [eV]	Retention time [min]
5OH‑2‑MEHTM	337	121^a)^	20	−22	6.3
		165	20	−18	
5cx‑1‑MEPTM	351	191^a)^	22	−12	6.3
		147	22	−22	
5cx‑2‑MEPTM	351	191^a)^	10	−12	6.7
		147	10	−24	
5OH‑1‑MEHTM	337	121^a)^	22	−24	6.7
		165	22	−18	
2‑MEHTM	321	277^a)^	22	−12	9.9
		233	22	−18	
1‑MEHTM	321	150^a)^	2	−14	10.1
		178	2	−16	
D_5_‑5cx‑1‑MEPTM	356	121	8	−28	6.3
D_5_‑5cx‑2‑MEPTM	356	191	14	−14	6.7
D_5_‑5OH‑1‑MEHTM	342	165	10	−18	6.7
D_5_‑2‑MEHTM	326	238	12	−16	9.9
D_5_‑1‑MEHTM	326	150	12	−16	10.1

a) Quantifier

**Fig.3:**
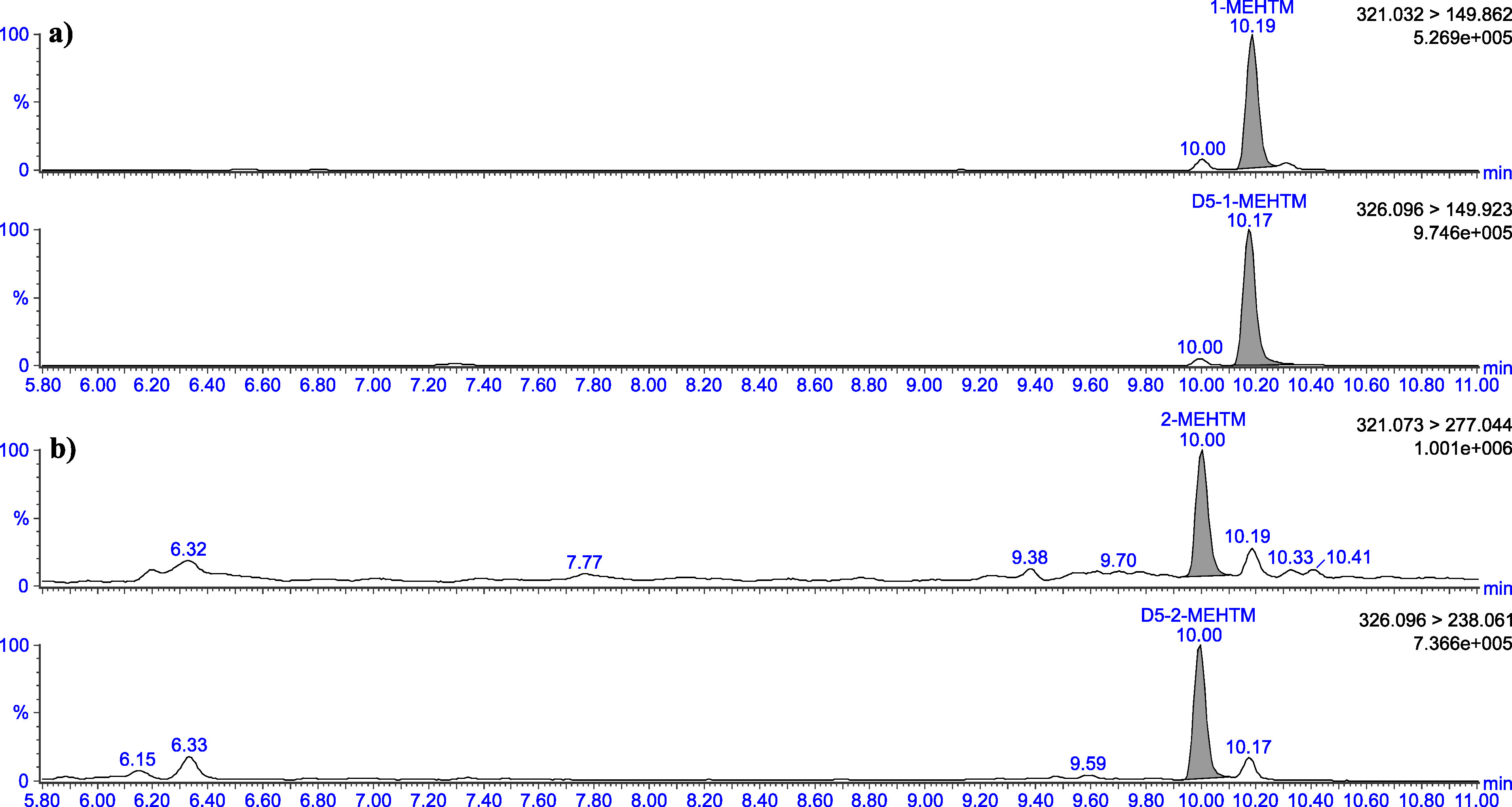
Chromatograms of a urine sample spiked with 2 μg 1‑MEHTM (a) and 2 μg 2‑MEHTM (b) per litre

**Fig.4:**
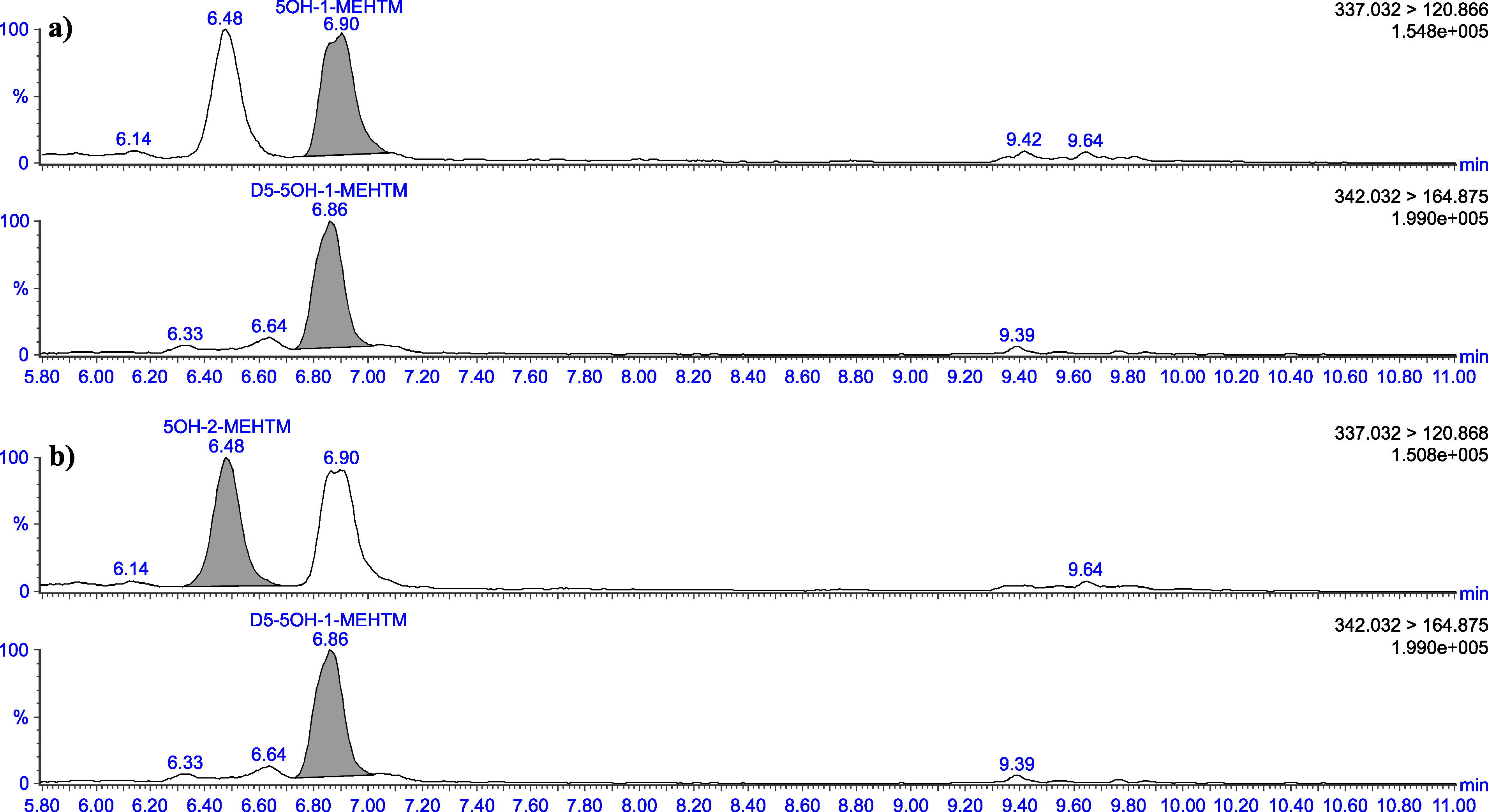
Chromatograms of a urine sample spiked with 2 μg 5OH‑1‑MEHTM (a) and 2 μg 5OH‑2‑MEHTM (b) per litre

**Fig.5:**
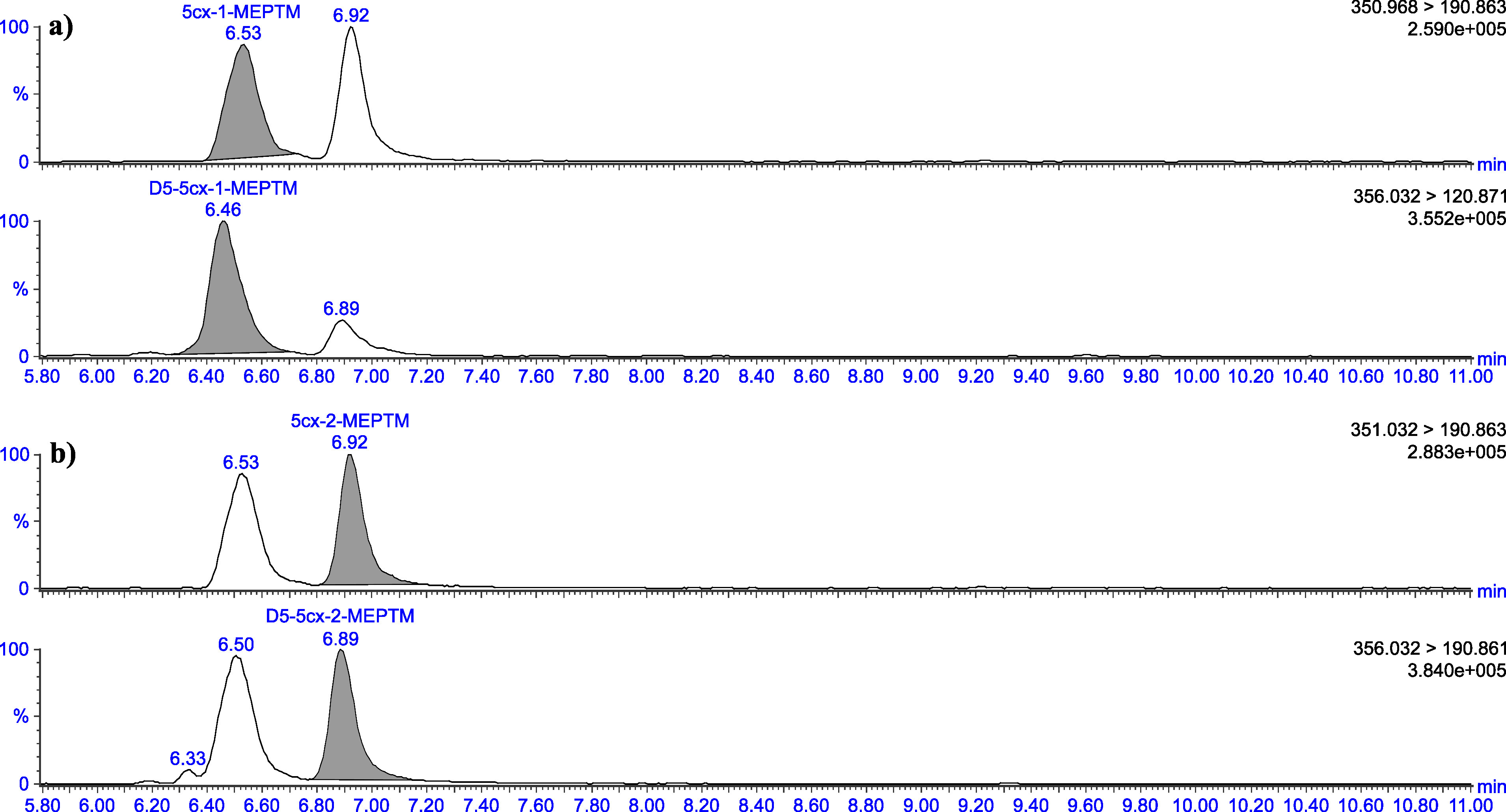
Chromatograms of a urine sample spiked with 2 μg 5cx‑1‑MEPTM (a) and 2 μg 5cx‑2‑MEPTM (b) per litre

**Fig.6:**
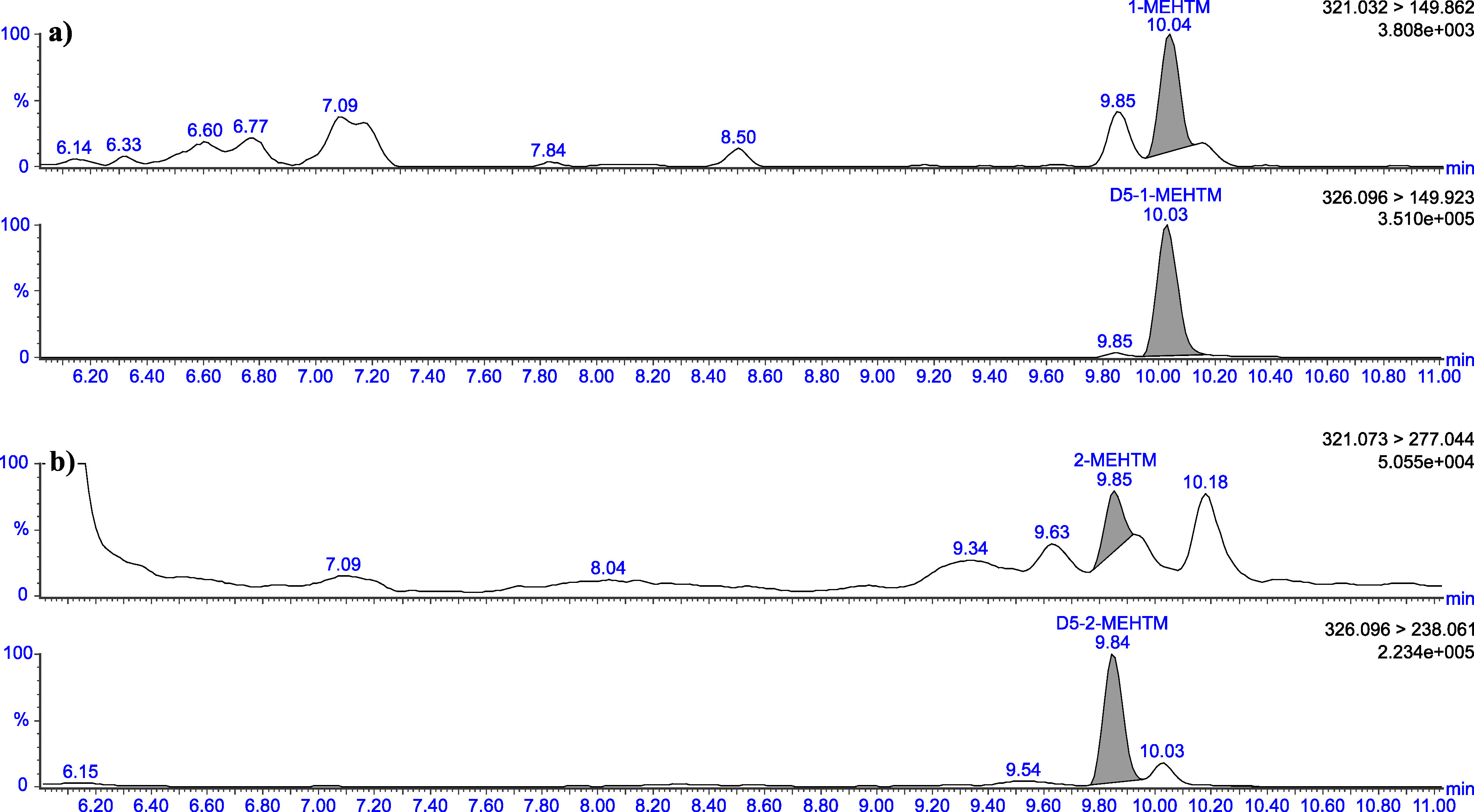
Chromatograms of a native urine sample with a concentration of 0.03 μg 1‑MEHTM/l (a) and 0.26 μg 2‑MEHTM/l (b)

**Fig.7:**
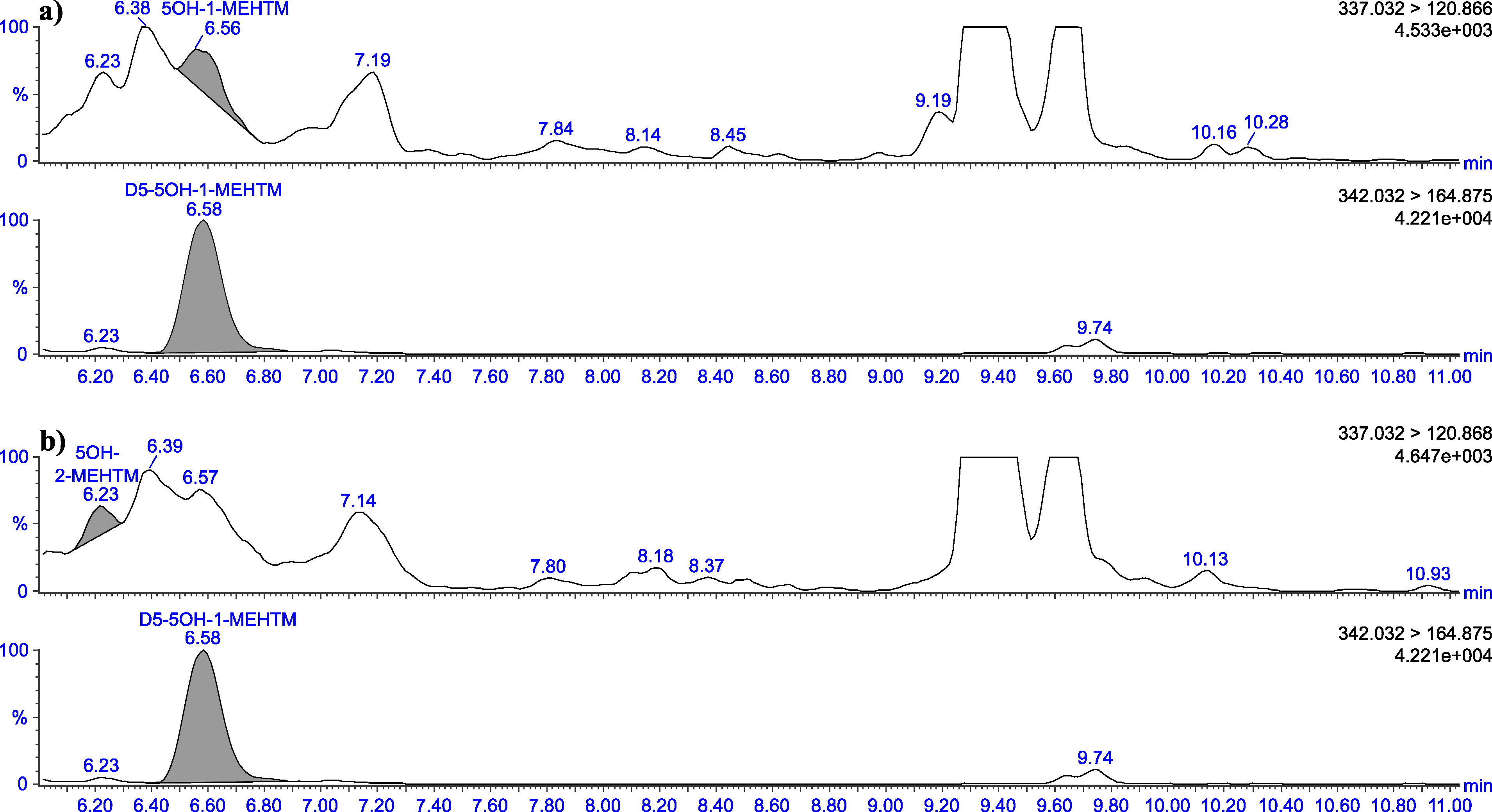
Chromatograms of a native urine sample with a concentration of 0.11 μg 5OH-1‑MEHTM/l (a) and 0.06 μg 5OH-2‑MEHTM/l (b)

**Fig.8:**
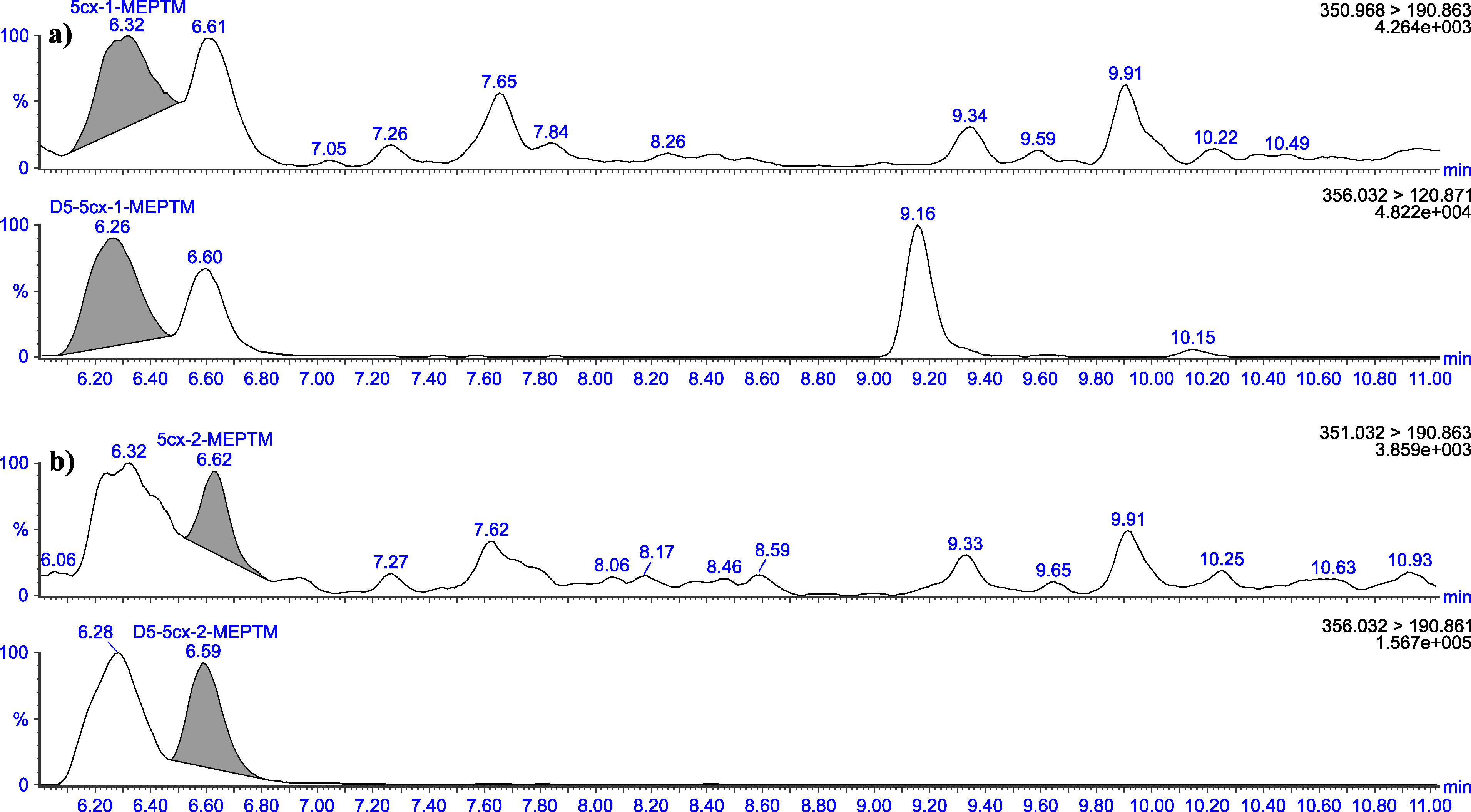
Chromatograms of a native urine sample with a concentration of 0.19 μg 5cx‑1‑MEPTM/l (a) and 0.04 μg 5cx‑2‑MEPTM/l (b)

The retention times given in Table 5 can only serve as a point of reference. The user of this method must ensure the separation performance of the column used and the resulting retention behaviour of the substances. Representative chromatograms are depicted in Figures 3 to 8.

## 8 Calibration

**Fig.9:**
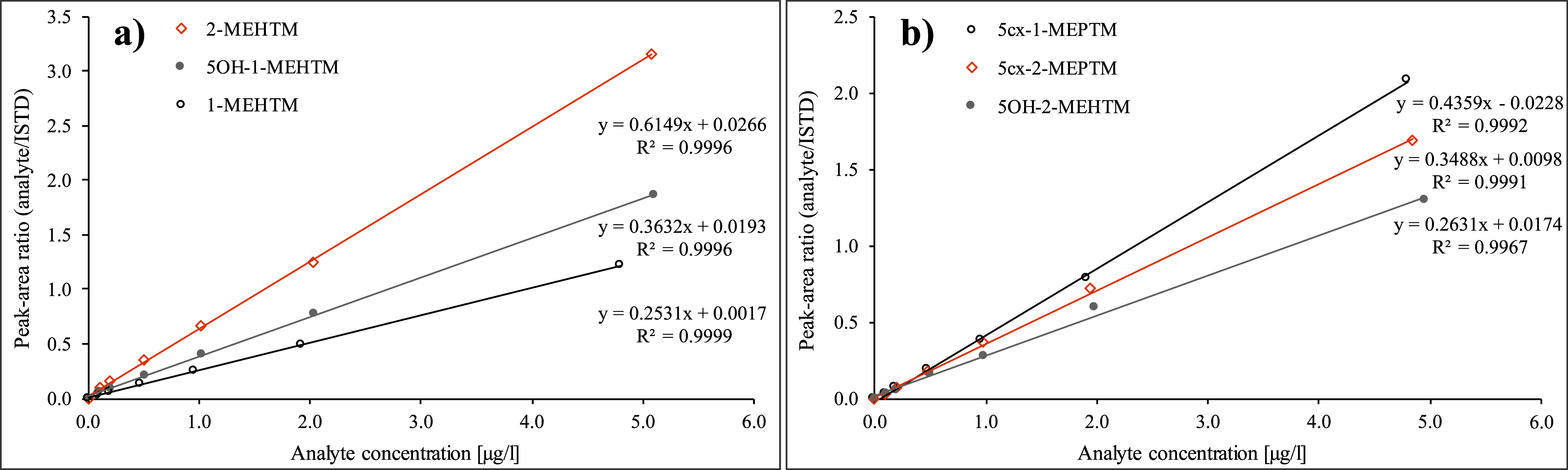
Representative calibration curves for the determination of TEHTM metabolites in urine

The calibration standards (see Section 4.5) are processed analogously to the urine samples according to Section 5 and analysed per Sections 6 and 7. The calibration curves are generated by plotting the quotients of the peak area of the analyte and the corresponding ISTD against the spiked concentrations of the individual calibration standard. D_5_‑5OH‑1‑MEHTM is used as the ISTD for the analyte 5OH‑2‑MEHTM. For all other analytes, structurally identical, isotope-labelled standards are available. Representative calibration curves are shown in Figure 9.

## 9 Calculation of analytical results

The analyte concentration of a sample is determined by dividing the peak area of the analyte by the peak area of the corresponding ISTD and inserting the quotient into the corresponding calibration function, yielding the analyte concentration in μg/l. Any reagent blanks which arise must be subtracted from the analytical results.

## 10 Standardisation and quality control

Quality assurance of the analytical results is carried out as stipulated in the guidelines of the *Bundesärztekammer*(German Medical Association) and in a general chapter published by the Commission (Bader et al. 2010; Bundesärzte­kammer 2023).

To test precision, at least two urine samples with known analyte concentrations are investigated as quality controls as part of each analytical run. Since such quality-control material is not commercially available, it must be prepared in the in-house laboratory. To this end, pooled urine is spiked with a defined amount of the analytes, aliquoted, and stored at −20 °C. For method validation, two control materials were prepared: Q_low_ and Q_high_ contained analyte concentrations of about 0.25 μg/l and 1.25 μg/l, respectively.

In addition, a reagent blank consisting of 1000 μl of ultra-pure water is measured as part of each analytical run. The reagent blank is processed and analysed analogously to the urine samples.

## 11 Evaluation of the method

The reliability of this method was confirmed by comprehensive validation as well as by replication and verification in a second, independent laboratory.

### 11.1 Precision

To determine within-day precision, the quality-control materials prepared according to Section 10 are repeatedly processed and measured in parallel. Tenfold determination of these urine samples yielded the precision data documented in Table 6.

**Tab.6:** Within-day precision for the determination of TEHTM metabolites in urine (n = 10)

Analyte	Spiked concentration [μg/l]	Standard deviation (rel.) ***s***_***w***_ **[%]**	Prognostic range ***u*** **[%]**
1‑MEHTM	0.24	3.0	6.8
	1.20	6.3	14.3
2‑MEHTM	0.25	4.3	9.7
	1.27	6.3	14.3
5OH‑1‑MEHTM	0.26	5.0	11.3
	1.28	4.0	9.0
5OH‑2‑MEHTM	0.25	8.1	18.3
	1.24	5.5	12.4
5cx‑1‑MEPTM	0.24	2.4	5.4
	1.20	6.0	13.6
5cx‑2‑MEPTM	0.24	4.8	10.9
	1.21	6.0	13.6

To determine day-to-day precision, the quality-control samples were processed and analysed on eight different days yielding the precision data documented in Table 7.

**Tab.7:** Day-to-day precision for the determination of TEHTM metabolites in urine (n = 8)

Analyte	Spiked concentration [μg/l]	Standard deviation (rel.) ***s***_***w***_ **[%]**	Prognostic range ***u*** **[%]**
1‑MEHTM	0.24	5.2	12.3
	1.20	6.6	15.9
2‑MEHTM	0.25	4.0	9.5
	1.27	7.3	17.3
5OH‑1‑MEHTM	0.26	5.0	11.8
	1.28	3.7	8.8
5OH‑2‑MEHTM	0.25	5.9	14.0
	1.24	2.7	6.3
5cx‑1‑MEPTM	0.24	6.0	14.2
	1.20	4.8	11.4
5cx‑2‑MEPTM	0.24	4.8	11.4
	1.21	5.6	13.2

### 11.2 Accuracy

The relative recovery of the analytes was calculated from the day-to-day precision data. The relative recoveries thus obtained are given in Table 8.

**Tab.8:** Relative recovery for the determination of TEHTM metabolites in urine (n = 8)

Analyte	Spiked concentration [μg/l]	Mean recovery (rel.) ***r*** **[%]**	Range **[%]**
1‑MEHTM	0.24	100	89.6–106
	1.20	99.0	93.7–113
2‑MEHTM	0.25	104	99.6–109
	1.27	96.8	90.1–111
5OH‑1‑MEHTM	0.26	98.9	91.4–107
	1.28	99.0	95.9–107
5OH‑2‑MEHTM	0.25	104	93.1–109
	1.24	109	104–112
5cx‑1‑MEPTM	0.24	105	95.9–115
	1.20	109	103–118
5cx‑2‑MEPTM	0.24	99.4	91.5–108
	1.21	100	88.9–109

### 11.3 Limits of detection and quantitation

The detection and quantitation limits were determined using the calibration-curve method according to DIN 32645 (DIN 2008). The equidistant ten-point calibration curves spanned the concentration range from 0.05–0.50 μg/l. The detection and quantitation limits obtained for the individual analytes are given in Table 9.

**Tab.9:** Limits of detection and quantitation for the determination of TEHTM metabolites in urine

Analyte	Detection limit [μg/l]	Quantitation limit [μg/l]
1‑MEHTM	0.01	0.04
2‑MEHTM	0.02	0.07
5OH‑1‑MEHTM	0.02	0.07
5OH‑2‑MEHTM	0.04	0.12
5cx‑1‑MEPTM	0.01	0.05
5cx‑2‑MEPTM	0.01	0.04

### 11.4 Analyte stability upon freezing/thawing and at room temperature

Storage tests were performed to check analyte stability during sample storage. To this end, urine was spiked with the analytes at a concentration of 80 μg/l and subjected to three freeze-thaw cycles. As an alternative, the spiked samples were stored in the dark at room temperature for four days. The stored samples were measured alongside freshly spiked samples in a single analytical run, whereby these were measured before and after each corresponding storage sample. The results of these measurements are shown in Table 10.

After four days of storage at room temperature, excellent recovery rates in the range of 95–110% were determined for all analytes. After completion of the three freeze-thaw cycles, the recoveries for 1-MEHTM and 2-MEHTM were acceptable at 87% and 85%, respectively. For the remaining analytes, the recoveries after three freeze-thaw cycles were very good. It is worth noting that the analyte concentration chosen for stability testing was in the range of potential high occupational exposure. If necessary, users of this method should check storage stability in a lower concentration range.

**Tab.10:** Stability of TEHTM metabolites in urine after sample storage

Analyte	Mean recovery (rel.) ***r*** **[%]**	
	after three freeze-thaw cycles	after four days at room temperature
1‑MEHTM	87.4	98.9
2‑MEHTM	85.4	101.0
5OH‑1‑MEHTM	91.4	94.6
5OH‑2‑MEHTM	106.5	110.3
5cx‑1‑MEPTM	109.4	99.4
5cx‑2‑MEPTM	103.9	98.3

### 11.5 Sources of error

The method described herein on the determination of TEHTM metabolites enables the reliable measurement of these parameters in urine. To maintain the quality of the method, however, various aspects must be considered.

The analytes of this method comprise groups of isomers which exhibit identical mass-spectrometric fragmentation. These groups are a) the secondary carboxy metabolites (5cx‑1‑MEPTM and 5cx‑2‑MEPTM) with the mass fragments *m/z* 147 and *m/z* 191, b) the secondary hydroxy metabolites (5OH‑1‑MEHTM and 5OH‑2‑MEHTM) with the mass fragments *m/z* 121 and *m/z* 165, and c) the primary monoester isomers (1‑MEHTM and 2‑MEHTM) with the mass fragments *m/z* 150, *m/z* 178, *m/z* 277, and *m/z* 233. A chromatographic base-line separation of the peaks is necessary for specific and selective identification of the individual isomers; in the method hereby presented, this separation was achieved using a low-dimension core-shell biphenyl column as well as by optimising the eluent gradient. The precolumn and the particle filter should be renewed after about 100 injections.

In some cases, it was observed that urinary matrix components interfered with the evaluation of the analytes 5OH‑1‑MEHTM, 5OH‑2‑MEHTM, and 5cx‑1‑MEPTM. This effect could be minimised by thorough flushing of the chromatographic system with organic solvents. Moreover, to avoid interference from the urine matrix, sufficient blank injections should be included in the measurement sequences, especially after urine samples with high creatinine concentrations.

Analyte concentrations above 10 μg/l may cause carryover effects. If these effects are observed, it is recommended to only work with concentrations in the range of 0–10 μg/l.

## 12 Discussion of the method

The method enables the reliable determination of a total of six TEHTM metabolites in urine. In order to conclusively identify all metabolites, the carboxy, hydroxy, and monoester isomers, which exhibit identical fragmentation, are base-line separated on the chromatographic column.

As previously published *in vitro* and *in vivo* studies have shown, after TEHTM absorption, a region‑selective ester cleavage takes place, yielding the monoester isomers 1‑MEHTM and 2‑MEHTM, which are also the main metabolites of TEHTM. Of the oxidatively formed secondary downstream products of these isomers, 5OH‑1‑MEHTM, 5OH‑2‑MEHTM, and 5cx‑1‑MEPTM represent the quantitatively most important metabolites *in vivo* (Höllerer et al. 2018 a).

In general, this method is characterised by easy sample workup. The obtained validation data demonstrate that the method is sensitive, reliable, and well-suited for the measurement of internal exposure in persons exposed to the plasticiser TEHTM. As TEHTM has, to date, primarily been used for medical products, the main route of exposure to this substance is the treatment of patients using medical products which contain TEHTM as a plasticiser.

Exposure of the general population, on the other hand, is considered to be low. In adolescents in Germany, the detection frequency of the urinary TEHTM metabolites determinable with this method was very low (Murawski et al. 2021). While 2‑MEHTM was present in concentrations above the limit of quantification in eleven of the 439 urine samples examined, 5cx‑2‑MEPTM could not be quantified in any of the samples. The remaining metabolites were detected in 0.23–0.68% of the urine samples. The cause of exposure could not be clearly determined in the individuals exposed to TEHTM.

**Instruments used** UPLC system (ACQUITY UPLC H‑Class System, Waters GmbH, Eschborn, Germany) with a quaternary pump (ACQ H‑Class QSM Plus, Waters GmbH, Eschborn, Germany), a binary pump (UPLC Binary SOL MGR, Waters GmbH, Eschborn, Germany), an autosampler (ACQ H‑Class FTN‑H Plus, Waters GmbH, Eschborn, Germany), and a column manager (ACQUITY UPLC CM‑A, Waters GmbH, Eschborn, Germany); triple-quadrupole mass spectrometer (Xevo TQ‑XS, Waters GmbH, Eschborn, Germany)
